# The mental internal friction scale (MIFS): development and psychometric validation of a culturally informed tripartite measure for Chinese university students

**DOI:** 10.3389/fpsyg.2026.1878188

**Published:** 2026-08-17

**Authors:** Liang Wang, Ya Li, Chunyang Wu

**Affiliations:** 1 School of Marxism, Henan University of Technology, Zhengzhou, China; 2 Student Mental Health Education Center, Henan University of Technology, Zhengzhou, China; 3 Department of Education, Henan Normal University, Xinxiang, China

**Keywords:** Chinese university students, measurement invariance, mental internal friction, psychometric validation, second-order hierarchical model

## Abstract

**Introduction:**

In the context of intense academic and employment competition, Chinese university students frequently experience a culturally specific psychological state termed “mental internal friction,” characterized by overthinking, emotional exhaustion, and behavioral paralysis. However, existing psychological tools lack the cultural sensitivity to capture this nuanced phenomenon.

**Methods:**

This study developed the Mental Internal Friction Scale (MIFS) and systematically evaluated its psychometric properties. An initial item pool was generated via literature review, semi-structured interviews, and expert evaluation. Using convenience sampling approach, 1214 Chinese university students were recruited and randomly split into two equal subsamples for Exploratory Factor Analysis (EFA) and Confirmatory Factor Analysis (CFA).

**Results:**

EFA extracted a robust tripartite structure: Cognitive Rumination (7 items), Emotional Exhaustion (6 items), and Behavioral Inhibition (5 items), explaining 76.24% of the total variance. CFA confirmed a second-order hierarchical structure (CFI = 0.949, TLI = 0.941, RMSEA = 0.071), with a general “mental internal friction” factor loading onto three distinguishable subdimensions. The MIFS demonstrated good internal consistency (Cronbach's α = 0.952), split-half reliability (0.919), and 4-week test-retest reliability (0.749). Convergent and criterion validities were supported by significant positive correlations with depression, anxiety, and rumination (*r* = 0.42 to 0.61, *p* < 0.01), alongside adequate average variance extracted (AVE > 0.50) and composite reliability (CR > 0.70). Furthermore, measurement invariance across gender, grade level, and family location was generally supported (ΔCFI ≤ 0.01, ΔRMSEA ≤ 0.015) in this sample.

**Discussion:**

The MIFS appears to be a reliable, valid, and culturally informed instrument that encapsulates the hierarchical cognitive-affective-behavioral mechanisms of mental friction, providing a useful empirical tool for targeted mental health screenings and interventions in Chinese higher education. Given that this is the first validation study, further research is needed to test its generalizability across diverse populations and educational contexts.

## 1 Introduction

### 1.1 Problem statement and context

As China's higher education system enters a phase of massification, contemporary university students face multifaceted challenges, including the intensified involution in academic pursuits and a severe employment landscape. Consequently, terms such as involution and anxiety have become highly prevalent in campus life (Ni et al., 2024). Although mental health issues among university students including depression and anxiety are well-documented in both Chinese and global contexts (Fu et al., 2023; World Health Organization, 2022; American College Health Association, 2023), these broad diagnostic categories do not fully capture the culturally specific psychological experiences of Chinese students. A substantial proportion of students remain in a suboptimal psychological state amidst complex macro-environmental shifts, and a more nuanced, culturally grounded understanding of their inner experiences is urgently needed. Against this backdrop, the concept of mental internal friction (MIF) has emerged as a widely used, culturally rooted term in contemporary Chinese society. It is conceptually defined as a psychological state wherein intense internal conflicts—characterized by excessive self-doubt, repetitive rumination, and catastrophic anticipation—lead to subjective fatigue and the maladaptive dissipation of psychological energy, ultimately resulting in diminished agency (Jing, 2023; Zheng, 2025). Crucially, mental internal friction is not merely a synonym for stress, anxiety, or depression, but represents a theoretically distinct construct with a unique cognitive-affective-behavioral signature. Stress predominantly emphasizes external stimuli or psychological stress responses (Lazarus and Folkman, 1984), whereas anxiety and depression focus largely on the immediate negative affect or its pathological variants (Beck, 1976). In contrast, mental internal friction specifically targets the dynamic process of internal cognitive wrestling, the continuous depletion of psychological energy, and the subsequent behavioral paralysis, reflecting a multidimensional syndrome that integrates cognitive conflict, resource depletion, and action inhibition. Specifically, MIF is defined by three core features: (1) intense internal cognitive conflict (e.g., excessive self-doubt, repetitive rumination, and catastrophic anticipation); (2) continuous depletion of psychological energy; and (3) behavioral paralysis. Unlike stress (external triggers), anxiety (negative emotions), or depression (pathological mood), MIF is defined by the dynamic interplay of cognitive conflict, resource depletion, and behavioral paralysis. It also diverges from rumination, which remains at the cognitive level, and from burnout, which stops at affective exhaustion.

### 1.2 Theoretical background and limitations of existing measures

Theoretically, mental internal friction is closely intertwined with ego depletion theory and rumination. Ego depletion theory posits that exerting self-control consumes limited psychological resources, thereby impairing subsequent self-regulation (Baumeister et al., 1998). Rumination refers to the repetitive cognitive focus on symptoms of distress and their potential causes and consequences (Nolen-Hoeksema and Morrow, 1991). Notably, under states of ego depletion, individuals demonstrate a significantly reduced capacity to regulate negative emotions (Hofmann et al., 2012).

Relevant risk factors include perfectionism and self-criticism (Hewitt and Flett, 1991), while rumination is a known predictor of the onset, maintenance, and relapse of depression (Nolen-Hoeksema et al., 2008). Conversely, Behavioral Activation theory emphasizes breaking the vicious cycle of withdrawal and internal friction through proactive action (Martell and Dimidjian, 2001). Additionally, some neuroscientific studies have linked the default mode network (DMN) to self-referential processing and rumination (Raichle et al., 2001; Buckner et al., 2008), suggesting a potential neural correlate that may inform future investigations into the mechanisms underlying MIF.

Although these theories provide a robust foundation for understanding mental internal friction, existing measurement tools fail to directly and comprehensively capture the core features of this multidimensional construct. For instance, the Ruminative Responses Scale (RRS; Nolen-Hoeksema and Morrow, 1991) focuses exclusively on repetitive negative thinking, omitting the components of energy depletion and behavioral inhibition. The Emotional Exhaustion subscale of the Maslach Burnout Inventory (MBI-EE; Maslach and Jackson, 1981) targets the depletion of affective resources but cannot capture cognitive internal wrestling. The Self-Control Scale (SCS; Tangney et al., 2004) assesses the level of self-control resources rather than the dynamic process of resource depletion. Similarly, the State-Trait Anxiety Inventory (STAI; Spielberger, 1983) lacks assessments of cognitive conflict and behavioral paralysis. Collectively, these instruments exhibit significant construct undercoverage and mechanism misalignment.

More critically, the predominant reliance on direct translations of Western measures inherently lacks cultural relevance. The mental internal friction experienced by Chinese university students is deeply embedded in local socio-cultural schemas. Influenced by collectivism and Confucian relational orientations, Chinese students tend to exhibit a more pronounced “relational self,” rendering them highly sensitive to peer evaluation and external expectations (Markus and Kitayama, 1991). They frequently suppress their authentic selves to maintain superficial harmony in interpersonal interactions, while driven by traditional beliefs such as “excellence in learning leads to officialdom” coupled with high achievement motivation, they are highly susceptible to excessive perfectionism. These cultural specificities dictate that internal friction among Chinese students often manifests as “high interpersonal involvement coupled with cognitive conflict and behavioral withdrawal”—a nuanced phenotype that existing Western-derived scales are ill-equipped to capture. The prerequisite for scientifically quantifying this psychological process is the development of a reliable, valid, and culturally informed assessment tool.

### 1.3 Research objectives and hypotheses

To bridge the aforementioned gaps, this study aimed to develop the Mental Internal Friction Scale (MIFS) for Chinese university students and to rigorously evaluate its psychometric properties. Grounded in the theoretical framework and conceptual definition outlined above, we hypothesized that mental internal friction is a multidimensional and hierarchical construct comprising three core dimensions: (1) **Cognitive Rumination**, reflecting pre-decisional or pre-action repetitive entanglement, overthinking, and catastrophic anticipation; (2) **Emotional Exhaustion**, reflecting the depletion of psychological energy, subjective fatigue, and somatic symptoms arising from internal psychological conflicts; and (3) **Behavioral Inhibition**, reflecting diminished agency and procrastination tendencies resulting from excessive introspection and worry.

This study was conducted in three phases. Phase 1 involved item development and content validity testing, wherein an initial item pool was generated through a literature review, semi-structured interviews, and expert evaluation. Phase 2 comprised Exploratory Factor Analysis (EFA) utilizing a random split-half subsample (*n* = 607) to explore the underlying dimensional structure of the scale. Phase 3 consisted of Confirmatory Factor Analysis (CFA) and further validity and reliability testing, employing the remaining half of the sample (*n* = 607) to confirm the construct validity, as well as to evaluate internal consistency, test-retest reliability, convergent validity, discriminant validity, and criterion-related validity.

## 2 Method

### 2.1 initial scale development and item pool construction

#### 2.1.1 Theoretical framework and qualitative basis

A bottom-up, exploratory approach was adopted to generate the theoretical framework. Building upon a systematic review of Ego Depletion Theory (Baumeister et al., 1998) and Rumination Theory (Nolen-Hoeksema and Morrow, 1991), grounded theory methodology was introduced to conceptualize the indigenous construct of mental internal friction. The theoretical framework utilized psychological resource depletion as the core thread, encompassing conflict processes at the cognitive level, exhaustion responses at the psychosomatic level, and executive dysfunction at the behavioral level.

To empirically validate and enrich this framework, semi-structured in-depth interviews were conducted with 53 university students. Purposive sampling was utilized to maximize demographic heterogeneity. Participants were recruited through multiple channels, including campus posters, online bulletin boards, We Chat groups, and referrals from student counselors, to ensure broad coverage across different grades, majors, and backgrounds. The sample comprised 15 freshmen, 18 sophomores, 12 juniors, 4 seniors, and 4 postgraduates; 17 males and 36 females; and spanned humanities/economics/management (*n* = 35), science/engineering (*n* = 14), and arts (*n* = 4). Each interview lasted approximately 40 to 60 min, with all sessions being audio-recorded with participants' prior informed consent and transcribed verbatim within 48 hours. The interview protocol focused on six key domains: (1) personal understanding of mental internal friction; (2) specific personal or peer experiences of internal friction; (3) specific manifestations; (4) primary triggers; (5) impacts on daily life; and (6) coping strategies employed.

Two trained independent coders performed three-phase coding (open coding—axial coding —selective coding) on the interview transcripts using NVivo 12 software. To resolve discrepancies and ensure coding reliability, the coders engaged in regular discussions to compare and negotiate coding outcomes. Through the constant comparative method, core categories were refined, ultimately establishing a tripartite theoretical architecture of mental internal friction among Chinese university students: Cognitive Rumination, Emotional Exhaustion, and Behavioral Inhibition.

#### 2.1.2 Generation of the initial item pool

The initial item pool was constructed via a dual-track approach combining deductive and inductive strategies.

First, utilizing a deductive approach, well-established measures with robust psychometric properties were systematically reviewed for theoretical reference, including the Ruminative Responses Scale (RRS; Nolen-Hoeksema and Morrow, 1991), the State-Trait Anxiety Inventory (STAI; Spielberger, 1983), and the Ego Depletion Source Scale (Tang et al., 2016). Notably, rather than directly translating or adopting items from these scales, the research team extracted core components semantically congruent with the operational definition of mental internal friction (e.g., “repetitive thinking about past mistakes,” “difficulty making decisions”). These components subsequently underwent contextual and cultural adaptation: Western daily scenarios were replaced with familiar campus contexts of Chinese university students, and the phrasing was adjusted to align with native Chinese linguistic conventions. This track yielded 17 initial items.

Second, utilizing an inductive approach, thematic analysis was conducted on the 53 interview transcripts to extract indigenous conceptualizations. The inter-coder agreement coefficient for this phase was 0.84, indicating good reliability. Discrepancies were resolved through discussion and adjudication by a third researcher. Culture-specific, high-frequency expressions and typical verbatim quotes were extracted from the interviews (e.g., “It feels like two little people fighting in my head,” “Always worrying about things that haven't happened yet,” “Afraid of disappointing others, so I don't dare to say no”). The research team then subjected these raw expressions to linguistic standardization: while strictly preserving their core semantics, they were reformulated into uniform declarative sentences from a first-person perspective, tailored to fit a 5-point Likert format. This track generated 10 initial items. Consequently, a preliminary item pool comprising 27 items was formed.

#### 2.1.3 Expert review and content validity evaluation

To rigorously ensure the content validity of the item pool, a modified Delphi method was employed. An expert panel consisting of six professionals with over 5 years of experience in university mental health education and psychological assessment was invited to independently and blindly evaluate the items against three primary criteria: (a) Relevance: whether the item's semantics accurately mapped onto the boundaries of its assigned theoretical dimension; (b) Representativeness: whether the phrasing aligned with the linguistic schemas and authentic psychological experiences of Chinese university students; and (c) Independence: whether there was conceptual overlap or semantic redundancy among items.

Based on the evaluation results, the Content Validity Ratio (CVR) was calculated for each item. Following the critical value criteria for a six-member panel, items with a CVR ≥ 0.99 were retained (Lawshe, 1975). The research team conducted two systematic iterations of the item pool based on these metrics: two items failing to meet the CVR threshold or exhibiting severe conceptual overlap were deleted (i.e., “My mind is always in a mess” and “I feel a pervasive psychological load”). Concurrently, retained items underwent refinement to reduce absolutism and enhance colloquial naturalness (e.g., changing “always” to “frequently”) to mitigate social desirability bias and defensive responding.

This process resulted in the final 25-item preliminary version of the Mental Internal Friction Scale (MIFS). The scale utilizes a 5-point Likert format (1 = completely disagree, 5 = completely agree). To accurately capture the state-like characteristics of mental internal friction, the instructions prompted respondents to rate the degree to which each statement applied to them “over the past two weeks.” Total scores range from 25 to 125, with higher scores indicating higher levels of mental internal friction. The complete initial item pool is detailed in Table 1.

**Table 1:** Initial item pool of the mental internal friction scale (MIFS).

Dimension	Item	Item wording
Cognitive rumination	A1	Even when nothing specific has happened, anxious or nervous thoughts repeatedly run through my mind.
	A2	My thoughts get uncontrollably trapped in repetitive loops over the same issue, making it hard to let go.
	A3	Before starting an important task, I often mentally rehearse various potential difficulties, leaving me exhausted before I even begin.
	A4	I repeatedly replay conversations with others, overanalyzing whether I said or did anything inappropriate.
	A5	I frequently experience conflicting thoughts internally, which leaves me feeling confused and uneasy.
	A6	I habitually deny myself, doubting my own abilities and worth.
	A7	I set high standards for myself; even when I perform well, I still feel it is not good enough.
	A8	I often find myself ruminating on the meaning of what I am currently doing (e.g., studying, activities), which leaves me feeling confused.
Emotional exhaustion	B9	I feel depressed or discouraged, and lack interest in many things.
	B10	Even after a night's rest, I still feel physically and mentally exhausted when I wake up the next day.
	B11	Over the past 2 weeks, I have frequently been troubled by negative emotions such as sadness, anger, and helplessness.
	B12	I feel prone to irritability or low mood, and find it hard to regulate my emotions.
	B13	When I see the “perfect lives” others display on social media, I feel dissatisfied with my own current situation.
	B14	I experience sleep disturbances, such as difficulty falling asleep, frequent dreaming, waking up easily, or sleepin lightly.
	B15	Even with sufficient sleep hours, I still feel groggy and lethargic during the day, struggling to recover my energy.
Behavioral inhibition	C16	I am highly concerned about how others perceive me and fear receiving negative evaluations.
	C17	I unconsciously compare myself to others, which leads to feelings of anxiety or inferiority.
	C18	I find it hard to resist the temptation of digital entertainment (e.g., smartphones, social media), causing my study or work time to be severely squeezed.
	C19	I make detailed study or work plans, but my actual completion rate is very low.
	C20	I am aware that I need to change certain unhealthy habits (e.g., staying up late, irregular diet), but I consistently fail to stick to them.
	C21	The massive influx of online information makes me feel restless, making it difficult to sustain focus on a single task for long periods.
C22	C22	I tend to escape from tasks I should be doing by engaging in unrelated activities, such as playing games, mindlessly scrolling, sleeping, or zoning out.
	C23	To avoid an imperfect outcome, I would rather not start at all, or I spend excessive time fixating on minor details.
	C24	When encountering difficulties in my studies or daily life, my first reaction is to withdraw or give up, rather than trying to find a solution.
	C25	I often give up trying new things or taking new opportunities simply out of the fear of not doing them well.

### 2.2 Participants and procedure

Using a convenience sampling approach, participants were recruited from a university in Henan Province to complete an online survey via the Wenjuanxing platform in October 2025. To control for common method bias and invalid responses, stringent data screening criteria were established: (1) completion time was less than the minimum reasonable time (calculated as total number of items × 2 seconds); (2) presence of highly patterned responding, such as selecting the same option for 10 or more consecutive items or exhibiting a “*Z*-shaped” response pattern; and (3) failure to pass an embedded attention-check item (e.g., “Please select “completely agree” for this item”).

A total of 1,246 questionnaires were collected. After excluding 32 invalid responses based on the aforementioned criteria, 1,214 valid questionnaires were retained, yielding an effective response rate of 97.43%. Using the random number generator in SPSS 26.0, the valid sample was randomly and equally divided into two subsamples: Sample A (*n* = 607) was designated for Exploratory Factor Analysis (EFA), and Sample B (*n* = 607) was utilized for Confirmatory Factor Analysis (CFA) and criterion-related validity testing. The sociodemographic characteristics of the two subsamples are presented in Table 2.

**Table 2:** Demographic characteristics of the participants (*N* = 1,214).

Variable	Category	*N*	Percentage (%)
Gender	Male	569	46.87
	Female	645	53.13
Grade level	Freshman	632	52.08
	Sophomore	305	25.12
	Junior	204	16.80
	Senior	73	6.01
Only child	Yes	219	18.04
	No	995	81.96
Family location	Large/medium-sized city	264	21.75
	Town	492	40.53
	Rural area	458	37.73
Political affiliation	CPC Member/probationary member	19	1.57
	CYL member	642	52.88
	Masses (non-partisan)	547	45.06
	Other	6	0.50
Had ever served as a student leader	Yes	910	74.96
	No	304	25.04
Academic programme	Humanities/social sciences	504	41.52
	STEM/sciences	710	58.48

### 2.3 Criterion measures

**Self-Rating Depression Scale (SDS; Zung, 1965)**. The SDS was used to assess the level of depressive symptoms over the past week. It consists of 20 items rated on a 4-point Likert scale (1 = none or a little of the time, 4 = most or all of the time), with 10 items reverse-scored. A standard score of 53 or above indicates the presence of depressive symptoms. The Cronbach's α for the scale in the current study was 0.869.

**Self-Rating Anxiety Scale (SAS; Zung**, **1971****)**. The SAS was utilized to evaluate anxiety symptoms over the past week. This 20-item measure shares the same 4-point scoring format as the SDS, with 5 items reverse-scored. A standard score of 50 or above suggests clinically significant anxiety. The Cronbach's α for the current sample was 0.814.

**Ruminative Responses Scale (RRS; Nolen-Hoeksema and Morrow**, **1991****)**. The Chinese version of the RRS, revised by Han and Yang (2009), was employed to assess the tendency to engage in repetitive, passive focus on symptoms of distress and their causes and consequences when experiencing negative affect. The scale comprises 22 items rated on a 4-point scale (1 = never, 4 = always), encompassing three dimensions: symptom-focused rumination, brooding, and reflection. Higher total scores indicate a stronger ruminative tendency. The Cronbach's α for this measure in the present study was 0.907.

### 2.4 Statistical analysis

Data collection and preliminary screening were managed using Microsoft Excel. Subsequent statistical analyses were conducted using SPSS 26.0 and AMOS 28.0. Specifically, SPSS was used for item analysis, Exploratory Factor Analysis (EFA), reliability testing (internal consistency and test-retest), and validity testing (convergent and criterion-related validity). AMOS was employed for Confirmatory Factor Analysis (CFA) and multi-group Confirmatory Factor Analysis to test measurement invariance across genders. The significance level for all statistical tests was set at *p* < 0.05 (two-tailed).

Prior to conducting Exploratory Factor Analysis (EFA) and Confirmatory Factor Analysis (CFA), we examined the underlying assumptions of factor analysis. First, missing data were inspected: no missing values were present in the final dataset, as the online survey platform (Wenjuanxing) enforced mandatory responses to all items before submission. Second, univariate outliers were identified by examining standardized *z*-scores for all items, with cases exceeding |z| > 3.29 considered as potential outliers (Tabachnick and Fidell, 2013). No extreme outliers were detected. Multivariate outliers were assessed using Mahalanobis distance with a conservative threshold of *p* < 0.001; no multivariate outliers were identified. Third, multivariate normality was evaluated using Mardia's coefficient of multivariate kurtosis, which yielded a value of 105.82 (critical ratio = 20.26), indicating that the multivariate normality assumption was not fully satisfied. Given the sensitivity of the maximum likelihood (ML) estimator to violations of multivariate normality, we employed the Bollen–Stine bootstrap procedure with 2,000 bootstrap samples to correct standard errors and obtain robust *p*-values for model fit evaluation (Bollen and Stine, 1992). This approach is recommended when multivariate normality assumptions are not met in CFA applications (Byrne, 2016).

## 3 Results

### 3.1 Item analysis

Item analysis was conducted using the total valid sample (*n* = 1,214). Employing both the critical ratio (CR) method via independent-samples *t*-tests and the corrected item-total correlation method, the results revealed that the CR values for all items reached the 0.001 significance level, and the corrected Pearson correlations between each item and the total scale score exceeded 0.30. Consequently, no items were deleted at this stage. These findings indicate that the initial items possessed high correlations with the target construct, adequately capturing the content domain of the measurement. Descriptive statistics for each item, including means, standard deviations, skew ness, and kurtosis, are presented in Table 3. The skewness and kurtosis values for all items were within acceptable ranges (|skewness| < 2, |kurtosis| < 7), suggesting that the item-level data were approximately normally distributed.

**Table 3:** Descriptive statistics for each item (*N* = 1,214).

Item	Mean	SD	Skewness	Kurtosis
A1	2.27	1.19	0.45	−0.91
A2	2.50	1.23	0.22	−1.07
A3	2.39	1.16	0.29	−0.95
A4	2.64	1.24	0.12	−1.09
A5	2.36	1.19	0.38	−0.94
A6	2.22	1.15	0.50	−0.78
A7	2.23	1.11	0.50	−0.66
A8	2.37	1.20	0.37	−0.96
B9	2.15	1.12	0.61	−0.64
B10	2.18	1.16	0.59	−0.70
B11	2.01	1.06	0.66	−0.60
B12	2.06	1.10	0.65	−0.65
B13	2.17	1.13	0.58	−0.63
B14	2.11	1.18	0.74	−0.52
B15	2.17	1.14	0.53	−0.84
C16	2.40	1.21	0.28	−1.12
C17	2.21	1.14	0.48	−0.84
C18	2.46	1.23	0.21	−1.18
C19	2.33	1.15	0.38	−0.91
C20	2.53	1.26	0.13	−1.29
C21	2.36	1.18	0.30	−1.06
C22	2.36	1.20	0.37	−0.96
C23	2.18	1.10	0.53	−0.63
C24	2.09	1.04	0.59	−0.50
C25	2.18	1.09	0.48	−0.78

### 3.2 Exploratory factor analysis (EFA)

First, Sample A (*n* = 607) was subjected to suitability tests for factor analysis. The results yielded a KMO value of 0.979 and a statistically significant Bartlett's test of sphericity (χ^2^ = 22180.073, *df* = 465, *p* < 0.001), indicating strong intercorrelations among items and excellent suitability for EFA.

**Figure 1:**
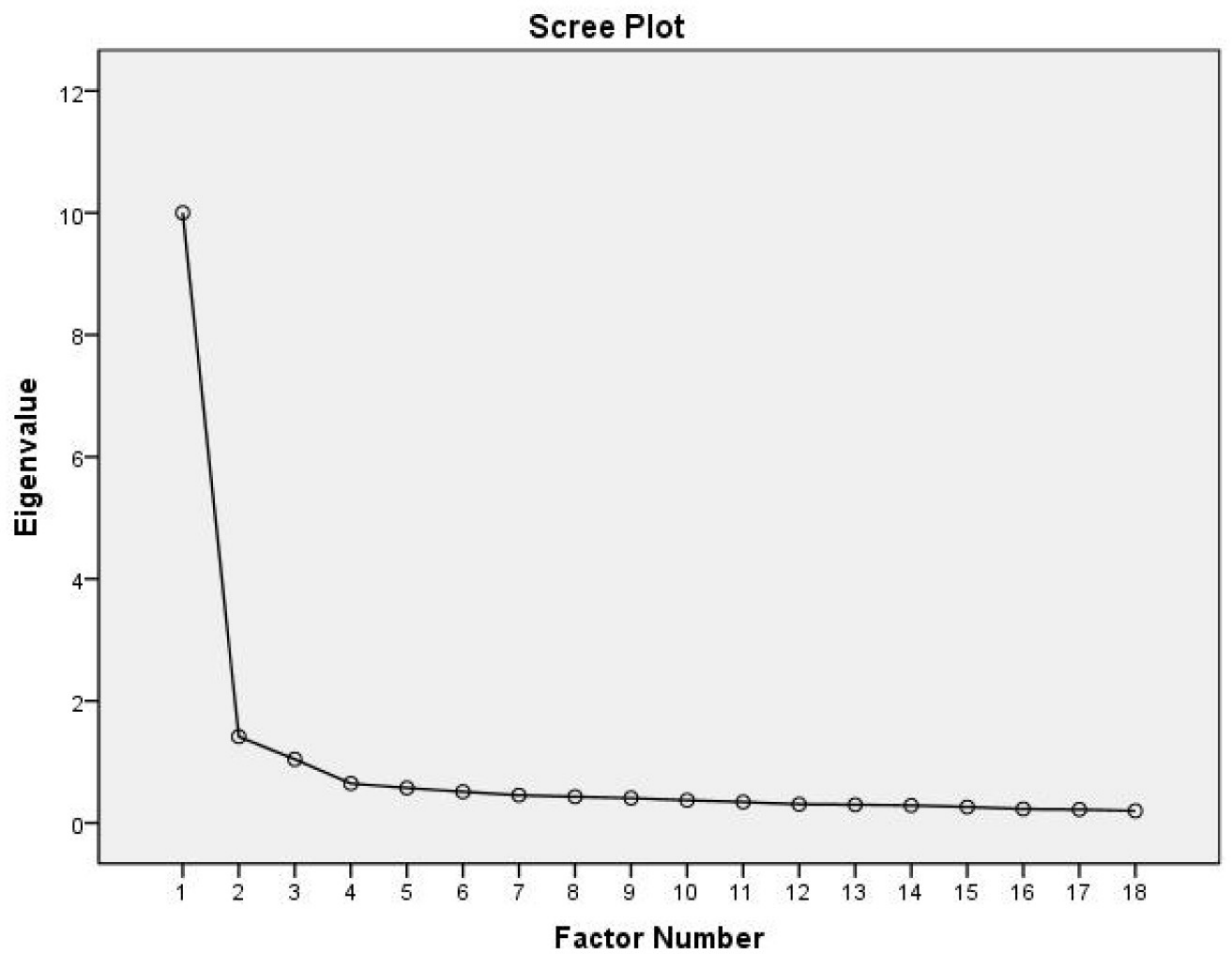
Scree plot of eigenvalues for the 18 items in EFA.

Principal axis factoring (PAF) with Promax oblique rotation was used for factor extraction. Initial factor retention was guided by the Kaiser–Guttman criterion (eigenvalues > 1). To cross-validate this decision, we additionally examined the scree plot (Cattell, 1966) and conducted parallel analysis (Horn, 1965) using Monte Carlo simulation with 100 randomly generated datasets at the 95th percentile. The scree plot revealed a clear inflection point after the third factor, and parallel analysis indicated that only the first three factors had eigenvalues exceeding the 95th percentile thresholds derived from random data. All three methods converged on a three-factor solution, providing robust support for factor retention. As shown in Figure 1, the eigenvalues leveled off after the third factor, suggesting that additional factors contributed little to the explained variance.

The retention and deletion of items were determined by the following comprehensive criteria: (1) a communality greater than 0.40; (2) a primary factor loading greater than 0.50; and (3) the absence of severe cross-loadings, specifically defined as a loading greater than 0.40 on a non-target factor, or an absolute difference between the loading on the target factor and the non-target factor of less than 0.20. An iterative process was applied: after each item deletion, the EFA was rerun until all remaining items strictly met the retention criteria.

The final solution retained 18 items after deleting 7 items (A8, B13, C16, C17, C23, C24, C25), which cleanly loaded onto three factors. Based on the theoretical framework and the specific content of the items loading on each factor, the three factors were labeled: Cognitive Rumination (7 items), Emotional Exhaustion (6 items), and Behavioral Inhibition (5 items). This three-factor structure accounted for 76.24% of the total variance. All retained items demonstrated factor loadings greater than 0.50 on their respective target factors, exhibiting a clear and simple structure (Table 4). The inter-factor correlations ranged from 0.74 to 0.75 (Table 5), suggesting that while the three factors are conceptually distinct, they share substantial underlying relationships. Overall, the three-factor model derived from the EFA demonstrated strong congruence with the initial theoretical conceptualization.

**Table 4:** The mental internal friction scale for chinese college students (MIFS).

Item	Item wording	Factor loadings			Commonalities
		Cognitive rumination	Emotional exhaustion	Behavioral inhibition	
A1	Even when nothing specific has doubting happened, anxious or nervous thoughts repeatedly run through my mind.	0.699	0.278	−0.075	0.572
A2	My thoughts get uncontrollably trapped in repetitive loops over the same issue, making it hard to let go.	0.792	0.125	−0.011	0.643
A3	Before starting an important task, I often mentally rehearse various potential difficulties, leaving me exhausted before I even begin.	0.687	0.092	0.157	0.505
A4	I repeatedly replay conversation with others, overanalyzing whether I said or did Anything inappropriate.	0.841	0.318	0.279	0.886
A5	I frequently experience conflicting thoughts internally, which leaves me feeling confused and uneasy.	0.762	0.008	0.171	0.610
A6	I habitually deny myself, my own abilities and worth.	0.588	0.403	−0.037	0.509
A7	I set high standards for myself; even When I perform well, I still feel it is not good enough.	0.626	0.281	−0.007	0.471
B9	I feel depressed or discouraged, and lack interest in many things.	0.154	0.836	−0.066	0.727
B10	Even after a night's rest, I still feel physically and mentally exhausted when I wake up the next day.	0.201	0.725	−0.020	0.566
B11	Over the past two weeks, I have frequently been troubled by negative emotions such as sadness, anger, and helplessness.	0.115	0.927	−0.132	0.889
B12	I feel prone to irritability or low mood, and find it hard to regulate my emotions.	0.122	0.836	−0.015	0.714
B14	I experience sleep disturbances, such as difficulty falling asleep, frequent dreaming, waking up easily, or sleeping lightly.	−0.038	0.733	0.148	0.560
B15	Even with sufficient sleep hours, I still feel groggy and lethargic during the day, struggling to recover my energy.	0.035	0.638	0.220	0.456
C18	I find it hard to resist the temptation of digital entertainment (e.g., smartphones, social media), causing my study or work time to be severely squeezed.	0.086	−0.050	0.894	0.809
C19	I make detailed study or work plans, but my actual completion rate is very low.	0.086	−0.050	0.802	0.651
C20	I am aware that I need to change certain unhealthy habits (e.g., staying up late, irregular diet), but I consistently fail to stick to them.	0.217	−0.037	0.801	0.704
C21	The massive influx of online information makes me feel restless, making it difficult to sustain focus on a single task for long periods.	0.143	−0.123	0.715	0.537
C22	I tend to escape from tasks I should be doing by engaging in unrelated activities, such as playing games, mindlessly scrolling, sleeping, or zoning out.	−0.031	0.187	0.747	0.594
Eigenvalues		3.825	5.521	4.832	
Cumulative variance contribution rate		76.24%			

**Table 5:** Inter–factor correlations of the mental internal friction scale.

Factor	1	2	3
1. Cognitive rumination	—		
2. Emotional exhaustion	0.74^**^	—	
3. Behavioral inhibition	0.75^**^	0.74^**^	—

^**^P < 0.01.

### 3.3 Confirmatory factor analysis

Using Sample B (*n* = 607), Confirmatory Factor Analysis (CFA) was conducted via AMOS 28.0 to cross-validate the factor structure derived from the EFA. To determine the optimal factor structure, a series of competing models were specified and evaluated: a single-factor model, a two-factor model, and the theoretically proposed three-factor model. As shown in Table 6, the three-factor model substantially outperformed the single-factor and two-factor models across all fit indices, indicating that the tripartite structure most accurately represents the underlying measurement properties of the scale.

**Table 6:** Comparison of fit indices for competing models.

Model	χ2/ df	GFI	AGFI	RMR	NFI	TLI	CFI	IFI	RMSEA
One–factor model(A + B + C)	11.167	0.721	0.647	0.089	0.811	0.715	0.801	0.825	0.130
Two–factor model(A + B, C)	7.150	0.814	0.762	0.068	0.880	0.880	0.895	0.895	0.101
Two–factor model(A, B + C)	9.146	0.749	0.680	0.080	0.846	0.840	0.860	0.861	0.116
Two–factor model(A + C, B)	7.608	0.801	0.747	0.073	0.872	0.871	0.887	0.887	0.104
Three–factor model (pre–modification)(A, B, C)	4.020	0.907	0.880	0.050	0.933	0.941	0.949	0.949	0.071
Three–factor model (post–modification)(A, B, C)	3.737	0.954	0.886	0.048	0.939	0.946	0.954	0.954	0.067
Second–order model	4.020	0.907	0.880	0.050	0.933	0.941	0.949	0.949	0.071
Bifactor model	6.089	0.893	0.849	0.159	0.907	0.900	0.921	0.921	0.092

A = cognitive rumination; B = emotional exhaustion; C = behavioral inhibition; “+” indicates combined factors.

Although the three-factor model outperformed the other competing models and met acceptable fit criteria, there remained room for improvement in its overall fit. Consequently, model modification was conducted iteratively, guided by modification indices (MI) in conjunction with theoretical considerations and item content. During this process, priority was given to adjusting error covariances with large MI values that possessed strong theoretical justifications. For instance, the error terms of Item 1 (e1) and Item 2 (e2), which share highly similar semantics, were allowed to correlate. Theoretically, while A1 focuses on non-specific intrusive thoughts and A2 focuses on event-specific obsessive rumination, both represent surface-level cognitive features within the rumination dimension. They are highly synergistic and mutually triggering during psychological processing; thus, correlating their residuals is theoretically well-grounded.

Re-estimation of the modified model revealed significant improvements across all fit indices. Compared to the initial three-factor model, the modified model demonstrated a decrease in χ^2^/ df from 4.020 to 3.737, and a reduction in RMSEA from 0.071 to 0.067, moving closer to the excellent threshold of 0.05. Furthermore, the GFI increased substantially from 0.907 to 0.954, reaching an excellent level.

Regarding the remaining paths with high MI values, the research team determined that they primarily represented cross-loadings or error covariances lacking theoretical support. Given that the modified model had already achieved adequate fit and further modifications lacked strong theoretical support, we did not pursue additional *post-hoc* adjustments to avoid the risk of over fitting, thereby compromising the model's generalizability to other independent samples (i.e., capitalization on chance).

Given the relatively high inter-factor correlations observed among the three dimensions (*r* = 0.74–0.75), we further tested a second-order hierarchical model, in which the three first-order factors (cognitive rumination, emotional exhaustion, and behavioral inhibition) loaded onto a single higher-order factor of “mental internal friction.” As presented in Table 6, the second-order model demonstrated satisfactory fit: χ^2^/ df = 4.020, GFI = 0.907, AGFI = 0.880, RMR = 0.050, NFI = 0.933, TLI = 0.941, CFI = 0.949, IFI = 0.949, RMSEA = 0.071. These fit indices were highly comparable to those of the modified three-factor correlated model (CFI = 0.954; RMSEA = 0.067), while the second-order model offered greater parsimony with fewer parameters (NPAR = 39 vs. more parameters in the modified three-factor model). The loadings of the three first-order factors on the second-order factor were all substantial: cognitive rumination (0.72), emotional exhaustion (0.90), and behavioral inhibition (0.97). Given its satisfactory fit, theoretical interpretability, and parsimony, the second-order model was adopted as the final representation of the factor structure, suggesting that mental internal friction is a unified higher-order construct that manifests through three distinguishable subdimensions.

To further examine whether the three dimensions reflect a general factor or represent distinct constructs, we additionally tested a bifactor model in which all 18 items loaded simultaneously on a general factor and on their respective specific factors, with the general factor and all specific factors specified as orthogonal to one another. As shown in Table 6, the bifactor model yielded χ^2^/ df = 6.089, CFI = 0.921, TLI = 0.909, RMSEA = 0.092, indicating marginal fit. Compared to the second-order model (χ^2^/ df = 4.020, CFI = 0.949, RMSEA = 0.071) and the modified three-factor correlated model (χ^2^/ df = 3.737, CFI = 0.954, RMSEA = 0.067), the bifactor model demonstrated considerably poorer fit. These results suggest that the three dimensions capture meaningful unique variance beyond a general factor, further supporting the adoption of the second-order model. Overall, the second-order hierarchical model demonstrated good fit and robust structural validity.

### 3.4 Reliability

Reliability analyses were performed on Sample B (*n* = 607). The MIFS demonstrated strong internal consistency, with a Cronbach's α of 0.952 for the total scale and a split-half reliability of 0.919. Subscale-level Cronbach's α values were 0.897 for Cognitive Rumination, 0.917 for Emotional Exhaustion, and 0.905 for Behavioral Inhibition. In addition, we calculated McDonald‘s omega (ω), which provides a more robust estimate of reliability under less stringent assumptions than α (Dunn et al., 2014). Omega values were 0.968 for the total scale and 0.918, 0.907, and 0.899 for the three subscales, respectively. The high consistency between the α and ω coefficients supports the satisfactory internal consistency of the MIFS across both total and subscale levels.

To evaluate temporal stability, a subsample of 60 participants was randomly selected to complete the MIFS again after a 4-week interval. The test-retest reliability of the total scale was 0.749 (*p* < 0.01), and the test-retest reliabilities for the three subscales ranged from 0.553 to 0.670 (*p* < 0.01), indicating moderate temporal stability over the 1-month period—consistent with the state-like nature of mental internal friction.

### 3.5 Criterion-related and convergent validity

Criterion-related validity was evaluated by examining the correlations between the MIFS and the SDS, SAS, and RRS (Table 7). The total score of the MIFS showed significant positive correlations with the SDS (*r* =0.46, *p* < 0.01), the SAS (*r* = 0.61, *p* < 0.01), and the RRS (*r* = 0.42, *p* < 0.01). These findings provide empirical evidence supporting the adequate criterion-related validity of the MIFS, confirming its robust associations with theoretically related constructs (i.e., depression, anxiety, and rumination).

**Table 7:** Correlation analysis between the mifs and criterion measures.

Measure	MIFS	SDS	SAS	RRS
MIFS	1	0.46^**^	0.61^**^	0.42^**^
SDS		1	0.70^**^	0.58^**^
SAS			1	0.67^**^
RRS				1

^**^P < 0.01.

The results of the convergent validity analysis (Table 8) revealed that the Average Variance Extracted (AVE) for all three subscales exceeded 0.50, and the Composite Reliability (CR) values were all greater than 0.70. These findings indicate that the scale demonstrates adequate convergent validity (Hair et al., 2010).

**Table 8:** Correlation matrix and discriminant validity assessment.

Subscale	1	2	3
1.Cognitive rumination	—		
2.Emotional exhaustion	0.74^**^	—	
3.Behavioral inhibition	0.75^**^	0.74^**^	—
AVE	0.604	0.616	0.653
√AVE	0.777	0.785	0.808
CR	0.911	0.908	0.903

^**^P < 0.01.

To assess discriminant validity, we adopted the Fornell–Larcker criterion (Fornell and Larcker, 1981), which requires that the square root of the AVE for each construct be greater than its correlations with all other constructs. As shown in Table 8, the square roots of the AVE for cognitive rumination (0.777), emotional exhaustion (0.785), and behavioral inhibition (0.808) were all larger than the inter-factor correlations among the three dimensions (ranging from 0.74 to 0.75). These results support the discriminant validity of the three-factor structure, indicating that although the three subscales are moderately to highly correlated, each captures a distinct aspect of mental internal friction.

### 3.6 Measurement invariance across subgroups

Using Sample B (*n* = 607), multi-group confirmatory factor analysis (MGCFA) was conducted via AMOS 28.0 to test the measurement invariance of the second-order hierarchical model across gender, grade, and family location (Table 9). For gender, the sample was divided into male (*n* = 360) and female (*n* = 247) groups. Grade level was dichotomized into lower-grade (freshmen and sophomores, *n* = 496) and upper-grade (juniors and seniors, *n* = 111) due to the relatively small sample size of seniors. Family location was categorized into urban (Large/Medium-sized city, *n* = 113), town (*n* = 238), and rural (*n* = 256) areas.

**Table 9:** Measurement invariance across gender, grade level, and family location.

Group	Model	χ^2^/ df	AIC	CFI	ΔCFI	TLI	ΔTLI	RMSEA	ΔRMSEA
Gender	Configural	2.683	876.032	0.940	—	0.935	—	0.053	—
	Metric	2.681	876.178	0.940	0.000	0.935	0.000	0.053	0.000
	Scalar	2.692	881.143	0.939	−0.001	0.935	0.000	0.053	0.000
Grade level	Configural	2.677	888.571	0.936	—	0.935	—	0.053	—
	Metric	2.703	880.027	0.940	0.004	0.934	0.001	0.053	0.000
	Scalar	2.692	881.143	0.939	−0.001	0.939	0.005	0.053	0.000
Family location	Configural	2.207	1,119.646	0.931	—	0.930	—	0.045	—
	Metric	2.212	1,122.727	0.931	0.000	0.930	0.000	0.045	0.000
	Scalar	2.159	1,101.197	0.931	0.000	0.933	0.003	0.044	−0.001

For each grouping variable, three progressively constrained models were estimated: configural invariance (equal form), metric invariance (equal factor loadings), and scalar invariance (equal item intercepts). Invariance was supported if changes in CFI and RMSEA between successive models remained within recommended thresholds (ΔCFI ≤ 0.01, ΔRMSEA ≤ 0.015; Chen, 2007). As shown in Table 9, configural, metric, and scalar invariance were supported across gender, grade level, and family location, with all ΔCFI and ΔRMSEA values falling within the recommended thresholds. These findings demonstrate that the second-order factor structure, factor loadings, and item intercepts of the MIFS are equivalent across gender, grade levels, and family location groups. This provides robust evidence that the measurement properties of the scale are invariant across these key demographic subgroups, supporting its broad applicability across diverse populations of Chinese university students. For academic programme, measurement invariance was not formally tested due to the combination of small sample size and limited response variability in the STEM group (*n*=104), which precluded reliable estimation of multi-group models (Kline, 2016). Notably, this issue appears to reflect restricted response variability among STEM students rather than sample size alone, as invariance was successfully established across grade level with a similarly sized subsample (*n* = 111).

## 4 Discussion

The present study aimed to develop a reliable and valid scale for measuring mental internal friction tailored to the actual context of Chinese university students. Grounded in the theoretical frameworks of Ego Depletion and Rumination, and supplemented by semi-structured interviews with 53 university students, a three-dimensional structural hypothesis for mental internal friction was proposed. Following a rigorous scale development procedure—including item generation, expert review, item analysis, exploratory factor analysis (EFA), confirmatory factor analysis (CFA), and reliability/validity testing—a formal 18-item scale encompassing three dimensions was established.

### 4.1 Theoretical structure and contributions of the scale

Structurally, both EFA and CFA supported a hierarchical three-factor structure, with a general second-order factor of mental internal friction loading onto three distinguishable subdimensions: “Cognitive Rumination,” “Emotional Exhaustion,” and “Behavioral Inhibition.” This structure is not only theoretically sound but also closely aligns with the actual psychological experiences of university students. Specifically, “Cognitive Rumination” points to the cognitive mechanisms underlying internal friction, characterized by unhelpful, repetitive entanglement and self-criticism. “Emotional Exhaustion” reflects the direct depletion of physical and mental resources, manifesting as persistent fatigue, emotional lability, and somatization. “Behavioral Inhibition” represents the impairment of volitional and behavioral functions, presenting as procrastination, distraction, and difficulties in self-control. The moderate to strong inter-correlations among these three dimensions (*r* = 0.74–0.75) outline a continuous process progressing from internal cognitive conflict to explicit behavioral inhibition, culminating in emotional exhaustion, thereby comprehensively capturing the multidimensional nature of mental internal friction.

Compared to existing measurement tools, the developed scale demonstrates significant integrative advantages and cultural relevance in its structural dimensions. Traditional anxiety or depression scales (e.g., SAS, SDS) primarily focus on negative emotional states and physiological arousal symptoms. While effective in assessing “physical and mental symptoms,” they often lack systematic consideration of “impaired behavioral functioning” and the underlying “cognitive mechanisms.” In contrast, the three-dimensional model constructed in this study transcends the limitations of a pure symptom-based approach. It not only covers the physiological and emotional indicators targeted by traditional scales but also innovatively incorporates the critical dimension of “Behavioral Inhibition.” This dimension delineates the “paralysis of will”—a state of “wanting to act but being unable to do so” experienced by contemporary Chinese university students facing conflicts between high expectations and realistic pressures within a hyper-competitive (“involution”) social context. This state-action discrepancy is difficult for singular, emotion-focused Western scales to capture. The Behavioral Inhibition dimension accurately reflects the “willpower depletion” described by Ego Depletion Theory, wherein individuals' executive functions are impaired due to the massive consumption of psychological resources caused by internal cognitive conflicts.

Furthermore, unlike Western scales that measure rumination solely as passive contemplation of negative emotions, the “Cognitive Rumination” dimension of this scale is deeply rooted in China's collectivistic culture and Confucian relational orientations, exhibiting unique cultural directivity. Rumination in Western contexts typically focuses on past losses or depressive symptoms; however, the rumination captured through our indigenous survey predominantly manifests as “catastrophic rehearsal of future difficulties” (e.g., anticipating upcoming hurdles) and “heightened sensitivity to interpersonal evaluation and collective harmony” (e.g., repetitively analyzing others' dialogues). By positioning these culturally specific “anticipatory rumination” and “interpersonally sensitive rumination” as the starting point, the scale links the complete chain of “cognitive rumination—emotional exhaustion—behavioral inhibition.” This multidimensional structure, extending from intrinsic cultural-psychological conflicts to explicit behavioral impediments, bridges the cultural blind spots of existing Western tools when assessing the “ineffective depletion of psychological resources” among Chinese students.

A critical question concerns whether the MIFS is culturally bound to the Chinese context or whether its applicability may extend to other collectivistic societies. The construct of mental internal friction emerged from culturally specific phenomena observed among Chinese university students, such as intense academic competition (“involution”), heightened sensitivity to interpersonal evaluation, and the internalization of Confucian values regarding achievement and social harmony. These features are likely shared, to varying degrees, by other East Asian societies with similar collectivistic orientations (e.g., Japan, Korea, and Vietnam). Nevertheless, the cross-cultural applicability of the MIFS beyond the Chinese context remains to be empirically tested. Specifically, the degree to which the three-dimensional structure and item content would be endorsed or interpreted similarly across different collectivistic cultures remains an open empirical question. Future research should therefore examine the cross-cultural measurement invariance of the MIFS in diverse samples beyond China before drawing firm conclusions about its generalizability.

### 4.2 Psychometric properties

Psychometrically, the scale demonstrated robust reliability and validity. The Cronbach's α for the total scale was 0.952, and the α coefficients for all subscales exceeded 0.897, indicating high internal consistency. This result is comparable to the reliability levels of the Ruminative Responses Scale (RRS) and ego depletion scales in existing literature, and slightly superior to some similar instruments. The test-retest reliability of the total scale was 0.749, with subscale reliabilities ranging from 0.553 to 0.670, demonstrating moderate temporal stability. Notably, the relatively lower test-retest reliability of the Behavioral Inhibition dimension (0.553) may be attributed to its strong state-like dependency; behavioral inhibition levels may fluctuate with recent life events and acute stress levels. Future research should further investigate the stability characteristics of this specific dimension.

The CFA results indicated that the second-order hierarchical model yielded good fit indices (χ^2^/df = 3.737, RMSEA = 0.067, CFI = 0.954) and significantly outperformed the single-factor and two-factor competing models, supporting the scale's structural validity. The convergent validity analysis revealed that the Average Variance Extracted (AVE) for all dimensions exceeded 0.50, and the Composite Reliability (CR) surpassed 0.70, further confirming the scale's adequate construct validity.

A noteworthy finding of this study is the moderate-to-strong correlations between MIF and depression, anxiety, and rumination (*r* = 0.42–0.61). These associations are consistent with theoretical expectations and provide support for convergent validity. However, they also raise understandable questions regarding the conceptual distinctiveness of the MIF construct.

To empirically evaluate this issue, we examined the proportion of shared variance between MIF and each related construct. The MIF total score shared only limited variance with these measures, with *r*^2^ values ranging from 0.18 (rumination) to 0.37 (anxiety), indicating that the majority of its variance (63%−82%) is not accounted for by these existing measures. These results suggest that MIF captures a substantial portion of unique variance beyond depression, anxiety, and rumination.

Beyond these quantitative analyses, it is also important to acknowledge that MIF overlaps conceptually with several established constructs. For instance, the Cognitive Rumination dimension shares conceptual ground with repetitive negative thinking (Ehring and Watkins, 2008); the Behavioral Inhibition dimension resembles procrastination (Steel, 2007) and psychological inflexibility (Kashdan and Rottenberg, 2010); and the core depletion process aligns with self-regulation failure (Baumeister et al., 1998). However, unlike these constructs—each of which captures a relatively narrow facet of dysfunction—MIF is defined by its integrative nature, simultaneously encompassing cognitive, emotional, and behavioral components within a culturally grounded framework. This integrative configuration positions MIF as a complementary assessment tool that captures a distinctive pattern of internal conflict and resource depletion not fully addressed by existing measures.

### 4.3 Theoretical contributions and practical implications

The primary theoretical contribution of this study lies in transcending the limitations of previous research that measured psychological distress from singular dimensions (e.g., anxiety, fatigue, or rumination). It integratively proposes and validates an operational definition and a hierarchical multidimensional measurement framework for the complex construct of “mental internal friction.” The developed scale not only responds to the widespread societal concern regarding “internal friction” but also provides a reliable and operable assessment tool for the empirical study of this concept, facilitating a transition from phenomenological discussion to scientific inquiry in this field.

On a practical level, this scale offers a valuable reference for mental health services in higher education institutions. Educators and counselors can utilize the scale for the screening and assessment of students' mental internal friction to identify individuals requiring intervention. The three dimensions of the scale also provide specific directions for targeted interventions: For “Cognitive Rumination,” Cognitive Behavioral Therapy (CBT) or mindfulness training could be introduced to help individuals break the cycle of repetitive overthinking; for “Emotional Exhaustion,” emotion regulation and stress management techniques could be integrated to help individuals restore psychophysiological balance; for “Behavioral Inhibition,” behavioral activation and goal-management strategies could be implemented to enhance behavioral execution.

While these findings suggest potential utility for research on mental health screening, caution should be exercised before recommending the MIFS for broad clinical screening applications. The present study established the reliability and structural validity of the scale, but predictive validity—specifically, whether MIFS scores can predict future mental health outcomes such as the onset or exacerbation of depression or anxiety—has not yet been established. At this stage, the MIFS is more appropriately conceptualized as a research instrument for assessing mental internal friction in university students, rather than a diagnostic or clinical screening tool. Future longitudinal research is needed to examine the prospective associations between MIFS and clinically relevant outcomes before the scale can be used in clinical or counseling settings.

Furthermore, the scale can serve as an outcome measure to evaluate the efficacy of relevant mental health curricula or intervention programs.

### 4.4 Limitations and future directions

First, there are limitations regarding sample representativeness. Although the sample size was adequate, the data were primarily collected from a single university in Henan Province. The geographical concentration and the homogeneity of the institution type fail to fully encompass university students across different economic development regions (e.g., East, Central, and West China) and different institutional tiers (e.g., key universities vs. regular universities, comprehensive vs. polytechnic institutions). Additionally, the use of convenience sampling inherently carries the risk of selection bias, as participants who were more accessible may systematically differ from the broader student population in terms of academic engagement or awareness of internal psychological experiences. Nevertheless, given that this is the first validation study of the MIFS, the single-site convenience sample provides an acceptable basis for preliminary psychometric evaluation; the primary goal at this stage is to establish the core psychometric properties and internal structure of the scale, rather than to claim immediate national representativeness. Future research should aim to expand the sampling scope using multi-center, stratified cluster sampling methods. Including student samples from diverse geographical regions and institutional types will aid in establishing more representative norms, thereby enhancing the ecological validity and generalizability of the scale.

Additionally, approximately 75% of the sample reported having ever served as student leaders during college. This is partly attributable to our broad operational definition of “student leader” (encompassing class committee members, student union officers, and student organization leaders across all grade levels) and the cumulative nature of such experience across multiple academic years. Nevertheless, students with prior leadership experience may differ from those without in terms of social engagement, organizational experience, and potentially their responses to self-report measures, which may limit the generalizability of the findings to the broader student population. Future studies should aim for more balanced samples to enhance representativeness.

Second, the absence of longitudinal data is a limitation. As a cross-sectional study, it is difficult to reveal the dynamic developmental characteristics of mental internal friction. Mental internal friction, as a process involving the continuous depletion of psychological resources, requires longitudinal research designs to thoroughly explore its developmental trajectories, fluctuation patterns, and dynamic relationships with external stressors. Moreover, whether MIFS scores can predict future mental health outcomes—such as the onset or exacerbation of depressive or anxiety symptoms—remains unknown. Future studies should employ longitudinal designs to track the trajectories of mental internal friction among university students and examine its influencing factors and predictive effects on mental health outcomes.

Third, the MIFS was developed and validated exclusively within a Chinese university student sample. Its applicability to other cultural contexts—including other collectivistic societies (e.g., Japan, Korea, Vietnam) and Western individualistic settings—has not been tested. Cross-cultural validation studies are needed to determine whether the factor structure, item functioning, and measurement invariance of the scale hold across different cultural groups.

Fourth, the reliance on self-report measures may introduce common method bias and social desirability effects, although we attempted to minimize these risks through procedural controls (e.g., anonymity, attention-check items, reverse-scored items). Future studies could incorporate multi-method assessments (e.g., peer reports, behavioral indicators, or ecological momentary assessment) to complement self-reported data and further reduce the influence of shared method variance. In addition, invariance across academic programmes could not be tested due to model non-convergence, likely attributable to the small STEM subsample (*n* = 104). Future research with larger and more balanced samples across academic disciplines is needed to systematically examine measurement invariance across different academic programmes.

## 5 Conclusion

The developed scale demonstrates a hierarchical factor structure comprising three subdimensions (cognitive rumination, emotional exhaustion, and behavioral inhibition) under a general second-order factor of mental internal friction. It serves as a valid instrument for assessing the level of mental internal friction among Chinese university students, offering a scientific basis for mental health education, early screening, and intervention efficacy evaluation in higher education institutions.

## Data Availability

The datasets presented in this study can be found in online repositories. The names of the repository/repositories and accession number(s) can be found in the article/supplementary material.
